# The top 100 most cited articles on total hip arthroplasty: a bibliometric analysis

**DOI:** 10.1186/s13018-019-1476-3

**Published:** 2019-12-04

**Authors:** Wenchao Zhang, Ning Tang, Xiaopeng Li, Daniel M. George, Guangxu He, Tianlong Huang

**Affiliations:** 1 Orthopaedic department, The Second Xiangya hospital of Central South University, Changsha, Hunan Province China; 2 Department of Applied Statistics, School of Mathematics and Statistics, Central South University, Changsha, Hunan Province China; 3 Royal Adelaide Hospital Adelaide, Adelaide, SA Australia

**Keywords:** Total hip arthroplasty, Total hip replacement, Bibliometric analysis

## Abstract

**Background:**

Over the past few decades, more and more articles about total hip arthroplasty have been published. We noticed, however, little is known about the characteristics and qualities of these studies.

**Methods:**

The databases of Web of Science Core Collection, BIOSIS Citation Index, MEDLINE, etc. were utilized for the identification of articles published from 1990 to May 2019. Total hip arthroplasty–related articles were identified, and the 100 most cited articles were selected for subsequent analysis of citation count, citation density (citations/article age), authorship, theme, geographic distribution, time-related flux, level of evidence, and network analysis.

**Results:**

The selected 100 articles were published mainly in the 1990s (46%) and 2000s (47%) with almost equal amount. Citations per article ranged from 994 to 191. Leading countries were the USA followed by Canada, England, and Sweden, all located in North America and Western Europe. The most highlighted study themes were postoperative thrombosis and surgical methods and materials. The most common level of evidence was level III (35%). The network analysis connoted that radiography, acetabulum, reoperation, and bone cement had a high degree of centrality in the 1990s, while cement had a high degree of centrality in the 2000s and 2010s.

**Conclusions:**

The time, area, and theme distribution of the top 100 most cited articles in the total hip arthroplasty have been thoroughly analyzed. It is noticeable that postoperative thromboembolism currently plays a major role in the field of total hip arthroplasty researches. However, most of them focus on the effectiveness of different treatments and drugs; little is known about its underlying mechanisms and influencing factors.

## Introduction

Total hip arthroplasty has evolved greatly in the last century. Since the early attempts to replace the hip joint by Sir John Charnley during the late nineteenth century, there have been many advances over the years. Over the years, much research has been done to explore and advance this therapeutic method. It has been demonstrated that total hip arthroplasty is an effective therapy for several hip diseases such as necrosis of the femoral head [1–3], hip osteoarthritis [4–6], and femoral neck fracture [7–9]. Amid hundreds of research topics in total hip arthroplasty, postoperative outcomes and surgical methods and materials remain the major focus.

There is a unique tool, called bibliometric analysis, for analyzing the qualities and characteristics of published articles. It was first published in the JAMA in 1987, and it has been widely used across diverse fields to evaluate and estimate the importance of published articles or trends in the research spotlight [10–12].

The aim of this study was to investigate the 100 most cited publications in the field of total hip arthroplasty, highlighting intellectual milestones in the field, and analyzing the qualities and characteristics of the most cited original papers over the past 30 years.

## Material and methods

### Search strategy and criteria

Articles were identified by searching the Web of Science Core Collection, BIOSIS Citation Index, KCI-Korean Journal Database, MEDLINE, Russian Science Citation Index, and SciELO Citation Index to retrieve all articles related to total hip arthroplasty. The search was performed by two independent researchers at the same time to enhance the search sensitivity. The search terms used were the following: “total hip arthroplast*” OR “total hip prosthesis implantation*” OR “total hip replacement*”.

**Fig. 1:**
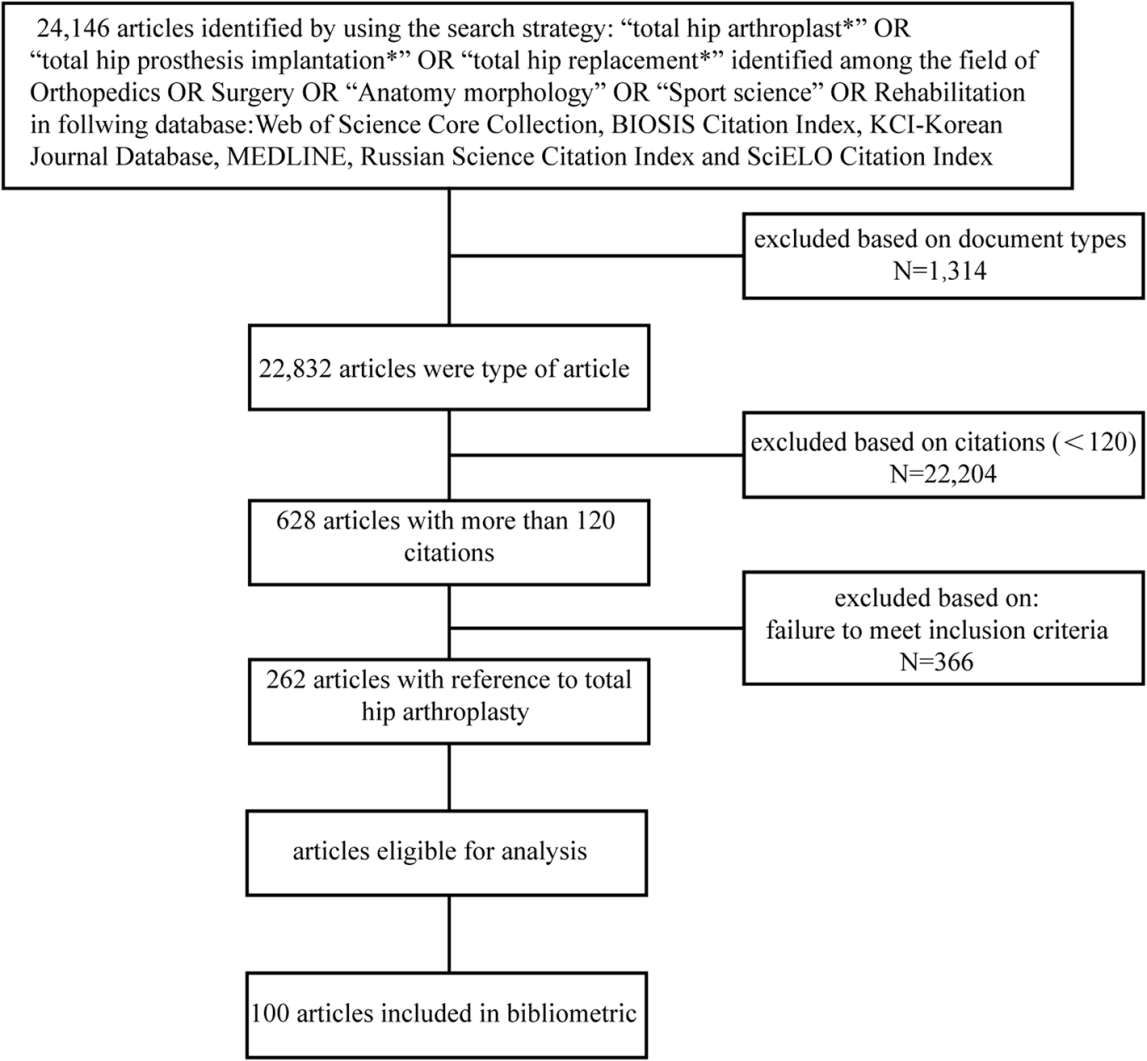
Flowchart illustrating the process of allocation of articles

The search was conducted in May 2019 and yielded a total of 24,146 results. Then, filtering the search results by “journal articles,” the results were 22,832. Only original articles were included. Thus, review articles, systematic reviews, meta-analyses, and guidelines were excluded while registry data were accepted. All articles cited less than 120 times were excluded to reduce the number of articles necessitating subsequent screening. This resulted in 628 articles included for analysis. Two independent investigators reviewed the title and the abstract of all included articles. Articles that met the following criteria were included: (1) basic study, animal study, and clinical trials related to any aspect of total hip arthroplasty; (2) the clinical therapeutic, prognostic, diagnostic, epidemiological studies of total hip arthroplasty; (3) the registry data in relevant institutions; (4) articles investigating materials or properties related to total hip arthroplasty. Disagreement between the two reviewers was discussed to reach an agreement. After the title and abstract review, there were 262 articles remaining. These articles were ranked in descending order of citations and the first 100 most cited articles were included in this analysis (Fig. 1).

### Data extraction

All articles were reviewed by two independent well-trained investigators. The following information was listed for all articles: the journal name, publication date, first author, year of publication, geographic origin, total number of citations of the article, overall citation rate (total citations/article age), research theme, and level of evidence (methodology has been described elsewhere [13]).

### Statistical analysis

The Shapiro-Wilk test was used to test the distribution of individual variables for normality. Normally distributed data are presented as mean ± standard deviation. Comparison between means was performed using one-way analysis of variance (ANOVA), and post-hoc testing was undertaken when necessary. Time-dependent trends were tested using the Mann-Kendall trend test. Correlation between variables was performed using the Spearman rank or Pearson tests. A *P* < 0.05 was considered to be statistically significant. Analysis was performed using IBM SPSS Statistics, Version 20.0. The Ucinet for windows, version 6.212 was used to perform the degree of centrality analysis [14].

## Results

**Fig. 2:**
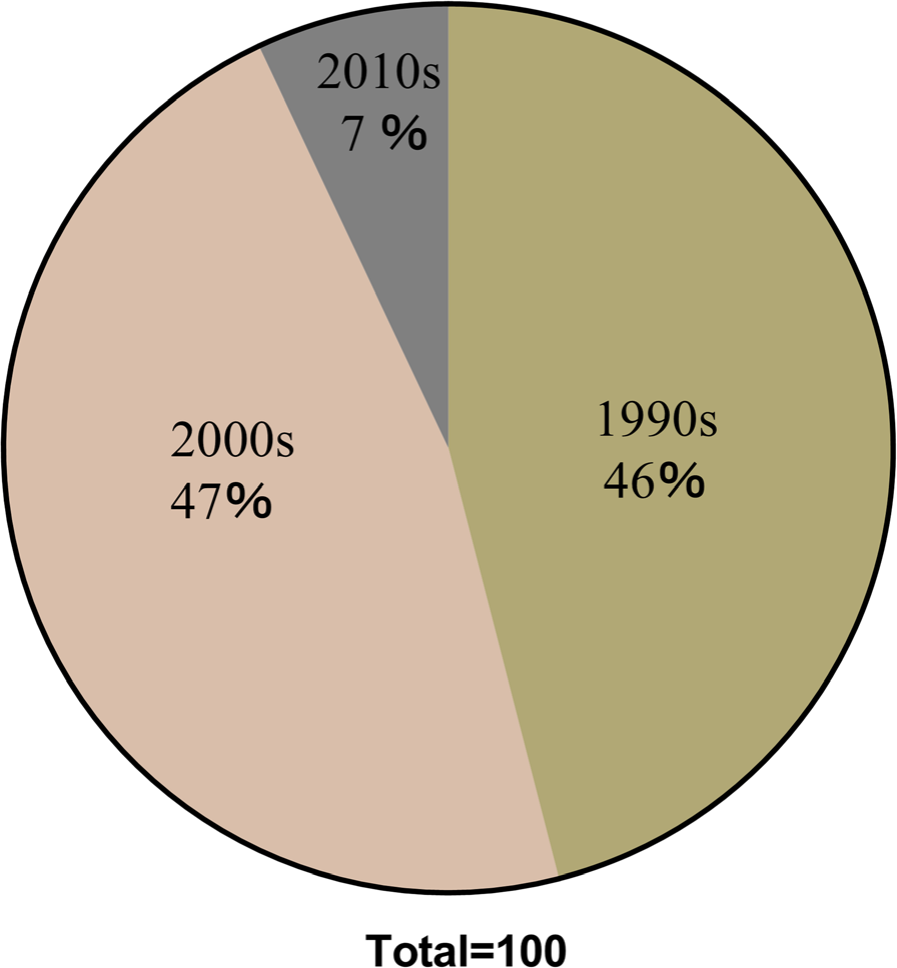
Time distribution of 100 top-cited articles in total hip arthroplasty. A majority of articles were published in the 1990s (46%, *n* = 46) and 2000s (47%, *n* = 47)

**Fig. 3:**
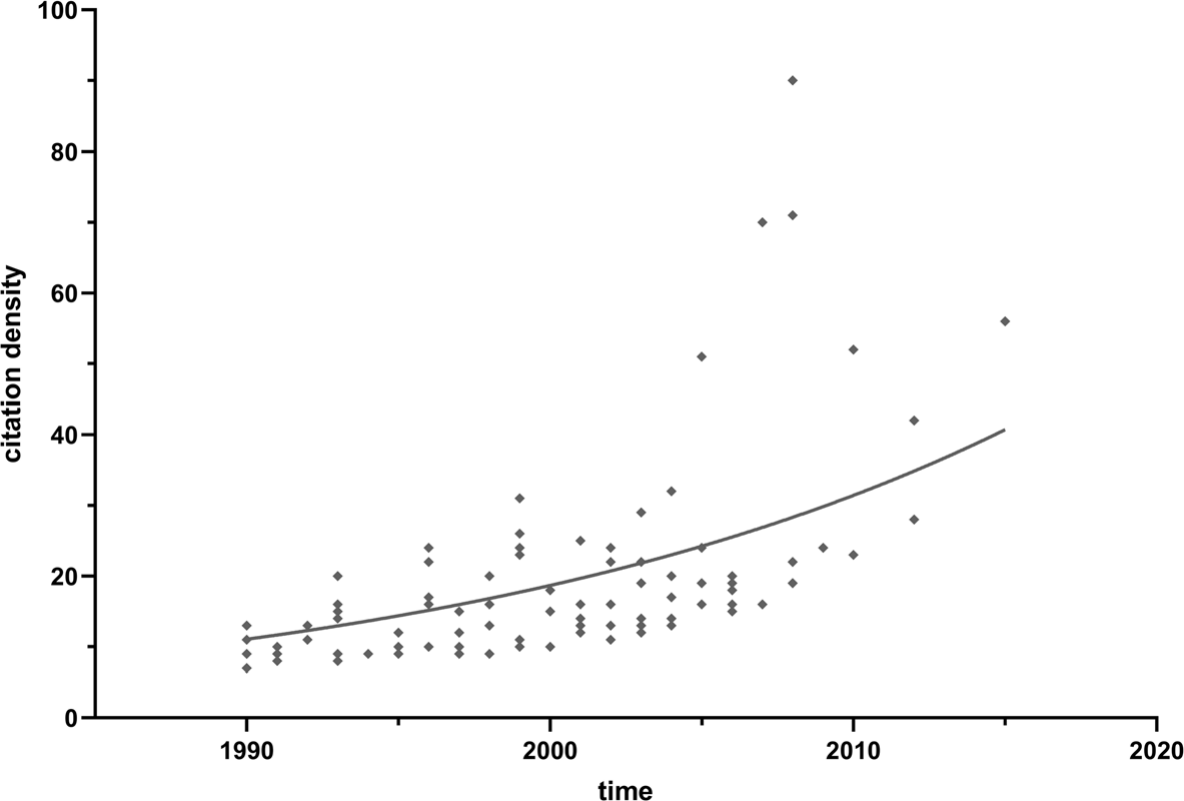
Time-dependent citation density trend. Mann-Kendall trend test showed an increasing trend between the citation density and the time (*P* = 5.854E−14)

We retrieved the 100 most cited articles on total hip arthroplasty and listed them in (Table 1). The number of citations ranges from 191 to 994, and a majority of articles were published in the 1990s (46%) and 2000s (47%). However, articles published since 2010 only accounted for 7% (Fig. 2). The year with the highest number of journals was 2003 (*n* = 8). The mean number of citations was 312 overall, 315 in the 1990s, 316 in the 2000s, and 269 in the 2010s. The Mann-Kendall trend test showed no time-dependent trend in the publication time of articles (*P* = 0.4162) but an increasing trend between the citation density and the time (*P* = 5.854E−14) (Fig. 3). The Spearman rank revealed a positive correlation between time and citation density (*r* = 0.695, *P* < 0.01). The Shapiro-Wilk test and the Kolmogorov-Smirnov test both indicated an abnormal distribution of the citation data.

**Table 1:** List of the 100 top-cited articles in total hip arthroplasty research

Rank	Article	Citations
1	Eriksson BI, Borris LC, Friedman RJ, Haas S, Huisman MV, Kakkar AK, Bandel TJ, Beckmann H, Muehlhofer E, Misselwitz F, Geerts W, Grp RS (2008) Rivaroxaban versus enoxaparin for thromboprophylaxis after hip arthroplasty. New England Journal of Medicine 358 (26):2765-2775. doi:10.1056/NEJMoa0800374	994
2	Eriksson BI, Dahl OE, Rosencher N, Kurth AA, van Dijk CN, Frostick SP, Prins MH, Hettiarachchi R, Hantel S, Schnee J, Bueller HR, Grp R-NS (2007) Dabigatran etexilate versus enoxaparin for prevention of venous thromboembolism after total hip replacement: a randomised, double-blind, non-inferiority trial. Lancet 370 (9591):949-956. doi:10.1016/s0140-6736(07)61445-7	834
3	Kakkar AK, Brenner B, Dahl OE, Eriksson BI, Mouret P, Muntz J, Soglian AG, Pap AF, Misselwitz F, Haas S, Investigators R (2008) Extended duration rivaroxaban versus short-term enoxaparin for the prevention of venous thromboembolism after total hip arthroplasty: a double-blind, randomised controlled trial. Lancet 372 (9632):31-39. doi:10.1016/s0140-6736(08)60880-6	777
4	Willert HG, Buchhorn GH, Fayyazi A, Flury R, Windler M, Koster G, Lohmann CH (2005) Metal-on-metal bearings and hypersensitivity in patients with artificial hip joints - A clinical and histomorphological study. Journal of Bone and Joint Surgery-American Volume 87A (1):28-36. doi:10.2106/JBJS.A.02039 pp	710
5	Bierbaum BE, Callaghan JJ, Galante JO, Rubash HE, Tooms RE, Welch RB (1999) An analysis of blood management in patients having a total hip or knee arthroplasty. Journal of Bone and Joint Surgery-American Volume 81A (1):2-10. doi:10.2106/00004623-199901000-00002	614
6	Schmied H, Kurz A, Sessler DI, Kozek S, Reiter A (1996) Mild hypothermia increases blood loss and transfusion requirements during total hip arthroplasty. Lancet (London, England) 347 (8997):289-292. doi:10.1016/s0140-6736(96)90466-3	562
7	McKellop H, Shen FW, Lu B, Campbell P, Salovey R (1999) Development of an extremely wear-resistant ultra high molecular weight polyethylene for total hip replacements. Journal of Orthopaedic Research 17 (2):157-167. doi:10.1002/jor.1100170203	522
8	Malchau H, Herberts P, Ahnfelt L (1993) Prognosis of total hip replacement in Sweden. Follow-up of 92,675 operations performed 1978-1990. Acta orthopaedica Scandinavica 64 (5):497-506. doi:10.3109/17453679308993679	520
9	Tsukayama DT, Estrada R, Gustilo RB (1996) Infection after total hip arthroplasty. A study of the treatment of one hundred and six infections. The Journal of bone and joint surgery American volume 78 (4):512-523. doi:10.2106/00004623-199604000-00005	506
10	Amstutz HC, Beaule PE, Dorey FJ, Le Duff MJ, Campbell PA, Gruen TA (2004) Metal-on-metal hybrid surface arthroplasty: Two to six-year follow-up study. Journal of Bone and Joint Surgery-American Volume 86A (1):28-39. doi:10.2106/00004623-200401000-00006	482
11	Spangehl MJ, Masri BA, O'Connell JX, Duncan CP (1999) Prospective analysis of preoperative and intraoperative investigations for the diagnosis of infection at the sites of two hundred and two revision total hip arthroplasties. Journal of Bone and Joint Surgery-American Volume 81A (5):672-683. doi:10.2106/00004623-199,905,000-00008	473
12	Mahomed NN, Barrett JA, Katz JN, Phillips CB, Losina E, Lew RA, Guadagnoli E, Harris WH, Poss R, Baron JA (2003) Rates and outcomes of primary and revision total hip replacement in the United States Medicare population. Journal of Bone and Joint Surgery-American Volume 85A (1):27-32. doi:10.2106/00004623-200301000-00005	471
13	Lassen MR, Gallus A, Raskob GE, Pineo G, Chen D, Ramirez LM, Investigators A- (2010) Apixaban versus Enoxaparin for Thromboprophylaxis after Hip Replacement. New England Journal of Medicine 363 (26):2487-2498. doi:10.1056/NEJMoa1006885	464
14	Fortin PR, Clarke AE, Joseph L, Liang MH, Tanzer M, Ferland D, Phillips C, Partridge AJ, Belisle P, Fossel AH, Mahomed N, Sledge CB, Katz JN (1999) Outcomes of total hip and knee replacement - Preoperative functional status predicts outcomes at six months after surgery. Arthritis and Rheumatism 42 (8):1722-1728. doi:10.1002/1529-0131(199908)42:8<1722::Aid-anr22>3.0.Co;2-r	461
15	Katz JN, Losina E, Barrett J, Phillips CB, Mahomed NN, Lew RA, Guadagnoli E, Harris WH, Poss R, Baron JA (2001) Association between hospital and surgeon procedure volume and outcomes of total hip replacement in the United States Medicare population. Journal of Bone and Joint Surgery-American Volume 83A (11):1622-1629. doi:10.2106/00004623-200111000-00002	442
16	White RH, Romano PS, Zhou H, Rodrigo J, Bargar W (1998) Incidence and time course of thromboembolic outcomes following total hip or knee arthroplasty. Archives of internal medicine 158 (14):1525-1531. doi:10.1001/archinte.158.14.1525	416
17	Berry DJ, Harmsen WS, Cabanela ME, Morrey BF (2002) Twenty-five-year survivorship of two thousand consecutive primary Charnley total hip replacements - Factors affecting survivorship of acetabular and femoral components. Journal of Bone and Joint Surgery-American Volume 84A (2):171-177. doi:10.2106/00004623-200202000-00002	414
18	Laupacis A, Bourne R, Rorabeck C, Feeny D, Wong C, Tugwell P, Leslie K, Bullas R (1993) The effect of elective total hip replacement on health-related quality of life. The Journal of bone and joint surgery American volume 75 (11):1619-1626. doi:10.2106/00004623-199311000-00006	412
19	Planes A, Vochelle N, Darmon JY, Fagola M, Bellaud M, Huet Y (1996) Risk of deep-venous thrombosis after hospital discharge in patients having undergone total hip replacement: double-blind randomised comparison of enoxaparin versus placebo. Lancet (London, England) 348 (9022):224-228. doi:10.1016/s0140-6736(96)01453-5	398
20	Schulte KR, Callaghan JJ, Kelley SS, Johnston RC (1993) The outcome of Charnley total hip arthroplasty with cement after a minimum twenty-year follow-up. The results of one surgeon. The Journal of bone and joint surgery American volume 75 (7):961-975. doi:10.2106/00004623-199307000-00002	395
21	Bergqvist D, Benoni G, Bjorgell O, Fredin H, Hedlundh U, Nicolas S, Nilsson P, Nylander G (1996) Low-molecular-weight heparin (enoxaparin) as prophylaxis against venous thromboembolism after total hip replacement. The New England journal of medicine 335 (10):696-700. doi:10.1056/nejm199609053351002	389
22	Mulroy RD, Jr., Harris WH (1990) The effect of improved cementing techniques on component loosening in total hip replacement. An 11-year radiographic review. The Journal of bone and joint surgery British volume 72 (5):757-760	382
23	Chang RW, Pellisier JM, Hazen GB (1996) A cost-effectiveness analysis of total hip arthroplasty for osteoarthritis of the hip. Jama 275 (11):858-865. doi:10.1001/jama.275.11.858	370
24	Greenfield S, Apolone G, McNeil BJ, Cleary PD (1993) The importance of co-existent disease in the occurrence of postoperative complications and one-year recovery in patients undergoing total hip replacement. Comorbidity and outcomes after hip replacement. Medical care 31 (2):141-154. doi:10.1097/00005650-199302000-00005	368
25	Mahomed NN, Liang MH, Cook EF, Daltroy LH, Fortin PR, Fossel AH, Katz JN (2002) The importance of patient expectations in predicting functional outcomes after total joint arthroplasty. Journal of Rheumatology 29 (6):1273-1279	368
26	Nilsdotter AK, Lohmander LS, Klassbo M, Roos EM (2003) Hip disability and osteoarthritis outcome score (HOOS) - validity and responsiveness in total hip replacement. Bmc Musculoskeletal Disorders 4. doi:10.1186/1471-2474-4-10	354
27	Bobyn JD, Mortimer ES, Glassman AH, Engh CA, Miller JE, Brooks CE (1992) Producing and avoiding stress shielding. Laboratory and clinical observations of noncemented total hip arthroplasty. Clinical orthopaedics and related research (274):79-96	341
28	Herberts P, Malchau H (2000) Long-term registration has improved the quality of hip replacement - A review of the Swedish THR Register comparing 160,000 cases. Acta Orthopaedica Scandinavica 71 (2):111-121. doi:10.1080/000164700317413067	337
29	Kennedy JG, Rogers WB, Soffe KE, Sullivan RJ, Griffen DG, Sheehan LJ (1998) Effect of acetabular component orientation on recurrent dislocation, pelvic osteolysis, polyethylene wear, and component migration. The Journal of arthroplasty 13 (5):530-534. doi:10.1016/s0883-5403(98)90052-3	333
30	Eriksson BI, Dahl OE, Buller HR, Hettiarachchi R, Rosencher N, Bravo ML, Ahnfelt L, Piovella F, Stangier J, Kalebo P, Reilly P, Grp BIS (2005) A new oral direct thrombin inhibitor, dabigatran etexilate, compared with enoxaparin for prevention of thromboembolic events following total hip or knee replacement: the BISTRO II randomized trial. Journal of Thrombosis and Haemostasis 3 (1):103-111. doi:10.1111/j.1538-7836.2004.01100.x	330
31	McCollum DE, Gray WJ (1990) Dislocation after total hip arthroplasty. Causes and prevention. Clinical orthopaedics and related research (261):159-170	323
32	Eriksson BI, Wille-Jorgensen P, Kalebo P, Mouret P, Rosencher N, Bosch P, Baur M, Ekman S, Bach D, Lindbratt S, Close P (1997) A comparison of recombinant hirudin with a low-molecular-weight heparin to prevent thromboembolic complications after total hip replacement. The New England journal of medicine 337 (19):1329-1335. doi:10.1056/nejm199711063371901	322
33	Maloney WJ, Jasty M, Harris WH, Galante JO, Callaghan JJ (1990) Endosteal erosion in association with stable uncemented femoral components. The Journal of bone and joint surgery American volume 72 (7):1025-1034. doi:10.2106/00004623-199072070-00011	308
34	O'Boyle CA, McGee H, Hickey A, O'Malley K, Joyce CR (1992) Individual quality of life in patients undergoing hip replacement. Lancet (London, England) 339 (8801):1088-1091. doi:10.1016/0140-6736(92)90673-q	306
35	Sehat KR, Evans RL, Newman JH (2004) Hidden blood loss following hip and knee arthroplasty - Correct management of blood loss should take hidden loss into account. Journal of Bone and Joint Surgery-British Volume 86B (4):561-565. doi:10.1302/0301-620x.86b4.14508	305
36	Phillips CB, Barrett JA, Losina E, Mahomed NN, Lingard EA, Guadagnoli E, Baron JA, Harris WH, Poss R, Katz JN (2003) Incidence rates of dislocation, pulmonary embolism, and deep infection during the first six months after elective total hip replacement. Journal of Bone and Joint Surgery-American Volume 85A (1):20-26. doi:10.2106/00004623-200301000-00004	304
37	Smith AJ, Dieppe P, Vernon K, Porter M, Blom AW, Natl Joint Registry England W (2012) Failure rates of stemmed metal-on-metal hip replacements: analysis of data from the National Joint Registry of England and Wales. Lancet 379 (9822):1199-1204. doi:10.1016/s0140-6736(12)60353-5	297
38	Widmer KH, Zurfluh B (2004) Compliant positioning of total hip components for optimal range of motion. Journal of Orthopaedic Research 22 (4):815-821. doi:10.1016/j.orthres.2003.11.001	294
39	Cooper HJ, Della Valle CJ, Berger RA, Tetreault M, Paprosky WG, Sporer SM, Jacobs JJ (2012) Corrosion at the Head-Neck Taper as a Cause for Adverse Local Tissue Reactions After Total Hip Arthroplasty. Journal of Bone and Joint Surgery-American Volume 94A (18):1655-1661. doi:10.2106/jbjs. K.01352	292
40	Zicat B, Engh CA, Gokcen E (1995) Patterns of osteolysis around total hip components inserted with and without cement. The Journal of bone and joint surgery American volume 77 (3):432-439. doi:10.2106/00004623-199,503,000-00013	292
41	Turpie AGG, Gallus AS, Hoek JA, Pentasaccharide I (2001) A synthetic pentasaccharide for the prevention of deep-vein thrombosis after total hip replacement. New England Journal of Medicine 344 (9):619-625. doi:10.1056/nejm200103013440901	292
42	Jones CA, Voaklander DC, Johnston DWC, Suarez-Almazor ME (2000) Health related quality of life outcomes after total hip and knee arthroplasties in a community based population. Journal of Rheumatology 27 (7):1745-1752	288
43	Lalor PA, Revell PA, Gray AB, Wright S, Railton GT, Freeman MA (1991) Sensitivity to titanium. A cause of implant failure? The Journal of bone and joint surgery British volume 73 (1):25-28	283
44	Jolles BM, Zangger P, Leyvraz PF (2002) Factors predisposing to dislocation after primary total hip arthroplasty - A multivariate analysis. Journal of Arthroplasty 17 (3):282-288. doi:10.1054/arth.2002.30286	279
45	Doorn PF, Campbell PA, Worrall J, Benya PD, McKellop HA, Amstutz HC (1998) Metal wear particle characterization from metal on metal total hip replacements: transmission electron microscopy study of periprosthetic tissues and isolated particles. Journal of biomedical materials research 42 (1):103-111. doi:10.1002/(sici)1097-4636(199810)42:1<103::Aid-jbm13>3.0.Co;2-m	275
46	Ogonda L, Wilson R, Archbold P, Lawlor M, Humphreys P, O’Brien S, Beverland D (2005) A minimal-incision technique in total hip arthroplasty does not improve early postoperative outcomes - A prospective, randomized, controlled trial. Journal of Bone and Joint Surgery-American Volume 87A (4):701-710. doi:10.2106/jbjs. D.02645	266
47	Martell JM, Berdia S (1997) Determination of polyethylene wear in total hip replacements with use of digital radiographs. The Journal of bone and joint surgery American volume 79 (11):1635-1641. doi:10.2106/00004623-199,711,000-00004	265
48	Hamadouche M, Boutin P, Daussange J, Bolander ME, Sedel L (2002) Alumina-on-alumina total hip arthroplasty - A minimum 18.5-year follow-up study. Journal of Bone and Joint Surgery-American Volume 84A (1):69-77. doi:10.2106/00004623-200,201,000-00011	264
49	Anthony PP, Gie GA, Howie CR, Ling RS (1990) Localised endosteal bone lysis in relation to the femoral components of cemented total hip arthroplasties. The Journal of bone and joint surgery British volume 72 (6):971-979	264
50	Matta JM, Shahrdar C, Ferguson T (2005) Single-incision anterior approach for total hip arthroplasty on an orthopaedic table. Clinical Orthopaedics and Related Research (441):115-124. doi:10.1097/01.blo.0000194309.70518.cb	260
51	Keating JF, Grant A, Masson N, Scott NW, Forbes JF, Scottish Orthopaedic Trials N (2006) Randomized comparison of reduction and fixation, bipolar hemiarthroplasty, and total hip arthroplasty - Treatment of displaced intracapsular hip fractures in healthy older patients. Journal of Bone and Joint Surgery-American Volume 88A (2):249-260. doi:10.2106/jbjs. E.00215	258
52	Woolson ST, Mow CS, Syquia JF, Lannin JV, Schurman DJ (2004) Comparison of primary total hip replacements performed with a standard incision or a mini-incision. Journal of Bone and Joint Surgery-American Volume 86A (7):1353-1358. doi:10.2106/00004623-200407000-00001	253
53	Eriksson BI, Borris L, Dahl OE, Haas S, Huisman MV, Kakkar AK, Misselwitz F, Kalebo P, Odixa-Hip Study I (2006) Oral, direct Factor Xa inhibition with BAY 59-7939 for the prevention of venous thromboembolism after total hip replacement. Journal of Thrombosis and Haemostasis 4 (1):121-128. doi:10.1111/j.1538-7836.2005.01657.x	253
54	Eriksson BI, Borris LC, Dahl OE, Haas S, Huisman MV, Kakkar AK, Muehlhofer E, Dierig C, Misselwitz F, Kalebo P, Invest OD-HS (2006) A once-daily, oral, direct Factor Xa inhibitor, rivaroxaban (BAY 59-7939), for thromboprophylaxis after total hip replacement. Circulation 114 (22):2374-2381. doi:10.1161/circulationaha.106.642074	251
55	Schinsky MF, Della Valle CJ, Sporer SM, Paprosky WG (2008) Perioperative testing for joint infection in patients undergoing revision total hip arthroplasty. Journal of Bone and Joint Surgery-American Volume 90A (9):1869-1875. doi:10.2106/jbjs. G.01255	247
56	Russotti GM, Harris WH (1991) Proximal placement of the acetabular component in total hip arthroplasty. A long-term follow-up study. The Journal of bone and joint surgery American volume 73 (4):587-592. doi:10.2106/00004623-199173040-00016	246
57	Geesink RG, Hoefnagels NH (1995) Six-year results of hydroxyapatite-coated total hip replacement. The Journal of bone and joint surgery British volume 77 (4):534-547	245
58	Soderman P, Malchau H (2001) Is the Harris hip score system useful to study the outcome of total hip replacement? Clinical Orthopaedics and Related Research (384):189-197	243
59	Jacobs JJ, Skipor AK, Black J, Urban Rm, Galante JO (1991) Release and excretion of metal in patients who have a total hip-replacement component made of titanium-base alloy. The Journal of bone and joint surgery American volume 73 (10):1475-1486. doi:10.2106/00004623-199173100-00005	243
60	Grammatopoulos G, Pandit H, Kwon YM, Gundle R, McLardy-Smith P, Beard DJ, Murray DW, Gill HS (2009) Hip resurfacings revised for inflammatory pseudotumour have a poor outcome. Journal of Bone and Joint Surgery-British Volume 91B (8):1019-1024. doi:10.1302/0301-620x.91b8.22562	242
61	Martell JM, Pierson RH, 3rd, Jacobs JJ, Rosenberg AG, Maley M, Galante JO (1993) Primary total hip reconstruction with a titanium fiber-coated prosthesis inserted without cement. The Journal of bone and joint surgery American volume 75 (4):554-571. doi:10.2106/00004623-199304000-00010	236
62	Morlock M, Schneider E, Bluhm A, Vollmer M, Bergmann G, Muller V, Honl M (2001) Duration and frequency of every day activities in total hip patients. Journal of Biomechanics 34 (7):873-881. doi:10.1016/s0021-9290(01)00035-5	234
63	Jones CA, Voaklander DC, Johnston WC, Suarez-Almazor ME (2001) The effect of age on pain, function, and quality of life after total hip and knee arthroplasty. Archives of Internal Medicine 161 (3):454-460. doi:10.1001/archinte.161.3.454	233
64	Pagnano W, Hanssen AD, Lewallen DG, Shaughnessy WJ (1996) The effect of superior placement of the acetabular component on the rate of loosening after total hip arthroplasty. The Journal of bone and joint surgery American volume 78 (7):1004-1014. doi:10.2106/00004623-199607000-00004	233
65	Korovessis P, Petsinis G, Repanti M, Repantis T (2006) Metallosis after contemporary metal-on-metal total hip arthroplasty - Five to nine-year follow-up. Journal of Bone and Joint Surgery-American Volume 88A (6):1183-1191. doi:10.2106/jbjs. D.02916	232
66	Mancuso CA, Salvati EA, Johanson NA, Peterson MG, Charlson ME (1997) Patients' expectations and satisfaction with total hip arthroplasty. The Journal of arthroplasty 12 (4):387-396. doi:10.1016/s0883-5403(97)90194-7	229
67	Urban RM, Jacobs JJ, Gilbert JL, Galante JO (1994) Migration of corrosion products from modular hip prostheses. Particle microanalysis and histopathological findings. The Journal of bone and joint surgery American volume 76 (9):1345-1359. doi:10.2106/00004623-199409000-00009	228
68	Colwell CW, Collis DK, Paulson R, McCutchen JW, Bigler GT, Lutz S, Hardwick ME (1999) Comparison of enoxaparin and warfarin for the prevention of venous thromboembolic disease after total hip arthroplasty - Evaluation during hospitalization and three months after discharge. Journal of Bone and Joint Surgery-American Volume 81A (7):932-940. doi:10.2106/00004623-199907000-00005	227
69	Stangier J, Eriksson BI, Dahl OE, Ahnfelt L, Nehmiz G, Stahle H, Rathgen K, Svard R (2005) Pharmacokinetic profile of the oral direct thrombin inhibitor dabigatran etexilate in healthy volunteers and patients undergoing total hip replacement. Journal of Clinical Pharmacology 45 (5):555-563. doi:10.1177/0091270005274550	227
70	Eriksson BI, Agnelli G, Cohen AT, Dahl OE, Mouret P, Rosencher N, Eskilson C, Nylander I, Frison L, Ogren M, Grp MIS (2003) Direct thrombin inhibitor melagatran followed by oral ximelagatran in comparison with enoxaparin for prevention of venous thromboembolism after total hip or knee replacement - The METHRO III study. Thrombosis and Haemostasis 89 (2):288-296	225
71	Tidermark J, Ponzer S, Svensson O, Soderqvist A, Tornkvist H (2003) Internal fixation compared with total hip replacement for displaced femoral neck fractures in the elderly - A randomised, controlled trial. Journal of Bone and Joint Surgery-British Volume 85B (3):380-388. doi:10.1302/0301-620x.85b3.13609	224
72	Engesaeter LB, Lie SA, Espehaug B, Furnes O, Vollset SE, Havelin LI (2003) Antibiotic prophylaxis in total hip arthroplasty - Effects of antibiotic prophylaxis systemically and in bone cement on the revision rate of 22,170 primary hip replacements followed 0-14 years in the Norwegian Arthroplasty Register. Acta Orthopaedica Scandinavica 74 (6):223. doi:10.1080/00016470310018135	223
73	Furnes O, Lie SA, Espehaug B, Vollset SE, Engesaeter LB, Havelin LI (2001) Hip disease and the prognosis of total hip replacements - A review of 53 698 primary total hip replacements reported to the Norwegian Arthroplasty Register 1987-99. Journal of Bone and Joint Surgery-British Volume 83B (4):579-586. doi:10.1302/0301-620x.83b4.11223	222
74	Kremers HM, Larson DR, Crowson CS, Kremers WK, Washington RE, Steiner CA, Jiranek WA, Berry DJ (2015) Prevalence of Total Hip and Knee Replacement in the United States. Journal of Bone and Joint Surgery-American Volume 97A (17):1386-1397. doi:10.2106/jbjs. N.01141	222
75	Lieberman JR, Dorey F, Shekelle P, Schumacher L, Thomas BJ, Kilgus DJ, Finerman GA (1996) Differences between patients' and physicians' evaluations of outcome after total hip arthroplasty. The Journal of bone and joint surgery American volume 78 (6):835-838. doi:10.2106/00004623-199606000-00005	219
76	Alberton GM, High WA, Morrey BF (2002) Dislocationm after revision total hip arthroplasty - An analysis of risk factors and treatment options. Journal of Bone and Joint Surgery-American Volume 84A (10):1788-1792. doi:10.2106/00004623-200210000-00008	219
77	Leyvraz PF, Bachmann F, Hoek J, Buller HR, Postel M, Samama M, Vandenbroek MD (1991) Prevention of deep vein thrombosis after hip replacement: randomised comparison between unfractionated heparin and low molecular weight heparin. BMJ (Clinical research ed) 303 (6802):543-548. doi:10.1136/bmj.303.6802.543	217
78	Shinar AA, Harris WH (1997) Bulk structural autogenous grafts and allografts for reconstruction of the acetabulum in total hip arthroplasty. Sixteen-year-average follow-up. The Journal of bone and joint surgery American volume 79 (2):159-168. doi:10.2106/00004623-199702000-00001	215
79	Mueck W, Borris LC, Dahl OE, Haas S, Huisman M, Kakkar AK, Kalebo P, Muelhofer E, Misselwitz F, Eriksson BI (2008) Population pharmacokinetics and pharmacodynamics of once- and twice-daily rivaroxaban for the prevention of venous thromboembolism in patients undergoing total hip replacement. Thrombosis and Haemostasis 100 (3):453-461. doi:10.1160/07-12-0714	213
80	Garbuz DS, Tanzer M, Greidanus NV, Masri BA, Duncan CP (2010) The John Charnley Award Metal-on-Metal Hip Resurfacing versus Large-diameter Head Metal-on-Metal Total Hip Arthroplasty A Randomized Clinical Trial. Clinical Orthopaedics and Related Research 468 (2):318-325. doi:10.1007/s11999-009-1029-x	208
81	Hailer NP, Garellick G, Karrholm J (2010) Uncemented and cemented primary total hip arthroplasty in the Swedish Hip Arthroplasty Register Evaluation of 170,413 operations. Acta Orthopaedica 81 (1):34-41. doi:10.3109/17453671003685400	208
82	Warwick D, Williams MH, Bannister GC (1995) Death and thromboembolic disease after total hip replacement. A series of 1162 cases with no routine chemical prophylaxis. The Journal of bone and joint surgery British volume 77 (1):6-10	207
83	Kim YH, Kim VE (1993) Uncemented porous-coated anatomic total hip replacement. Results at six years in a consecutive series. The Journal of bone and joint surgery British volume 75 (1):6-13	207
84	Sochart DH (1999) Relationship of acetabular wear to osteolysis and loosening in total hip arthroplasty. Clinical Orthopaedics and Related Research (363):135-150	206
85	Baker RP, Squires B, Gargan MF, Bannister GC (2006) Total hip arthroplasty and hemiarthroplasty in mobile, independent patients with a displaced intracapsular fracture of the femoral neck - A randomized, controlled trial. Journal of Bone and Joint Surgery-American Volume 88A (12):2583-2589. doi:10.2106/jbjs. E.01373	206
86	Mulroy WF, Estok DM, Harris WH (1995) Total hip arthroplasty with use of so-called second-generation cementing techniques. A fifteen-year-average follow-up study. The Journal of bone and joint surgery American volume 77 (12):1845-1852. doi:10.2106/00004623-199512000-00008	206
87	Bertin KC, Rottinger H (2004) Anterolateral mini-incision hip replacement surgery - A modified Watson-Jones approach. Clinical Orthopaedics and Related Research (429):248-255. doi:10.1097/01.blo.0000150294.81825.8c	205
88	Hull RD, Raskob GE, Gent M, McLoughlin D, Julian D, Smith FC, Dale NI, Reed-Davis R, Lofthouse RN, Anderson C (1990) Effectiveness of intermittent pneumatic leg compression for preventing deep vein thrombosis after total hip replacement. Jama 263 (17):2313-2317. doi:10.1001/jama.263.17.2313	203
89	Joshi AB, Porter ML, Trail IA, Hunt LP, Murphy JC, Hardinge K (1993) Long-term results of Charnley low-friction arthroplasty in young patients. The Journal of bone and joint surgery British volume 75 (4):616-623	203
90	Santavirta S, Hoikka V, Eskola A, Konttinen YT, Paavilainen T, Tallroth K (1990) Aggressive granulomatous lesions in cementless total hip arthroplasty. The Journal of bone and joint surgery British volume 72 (201):980-984	201
91	Karlson EW, Mandl LA, Aweh GN, Sangha O, Liang MH, Grodstein F (2003) Total hip replacement due to osteoarthritis: The importance of age, obesity, and other modifiable risk factors. American Journal of Medicine 114 (2):93-98. doi:10.1016/s0002-9343(02)01447-x	201
92	Espehaug B, Engesaeter LB, Vollset SE, Havelin LI, Langeland N (1997) Antibiotic prophylaxis in total hip arthroplasty. Review of 10,905 primary cemented total hip replacements reported to the Norwegian arthroplasty register, 1987 to 1995. The Journal of bone and joint surgery British volume 79 (200):590-595. doi:10.1302/0301-620x.79b4.7420	200
93	Pollard TCB, Baker RP, Eastaugh-Waring SJ, Bannister GC (2006) Treatment of the young active patient with osteoarthritis of the hip - A five- to seven-year comparison of hybrid total hip arthroplasty and metal-on-metal resurfacing. Journal of Bone and Joint Surgery-British Volume 88B (5):592-600. doi:10.1302/0301-620x.88b5.17354	200
94	Jaramaz B, DiGioia AM, 3rd, Blackwell M, Nikou C (1998) Computer assisted measurement of cup placement in total hip replacement. Clinical orthopaedics and related research (354):70-81. doi:10.1097/00003086-199809000-00010	197
95	Blomfeldt R, Tornkvist H, Eriksson K, Soderqvist A, Ponzer S, Tidermark J (2007) A randomised controlled trial comparing bipolar hemiarthroplasty with total hip replacement for displaced intracapsular fractures of the femoral neck in elderly patients. Journal of Bone and Joint Surgery-British Volume 89B (2):160-165. doi:10.1302/0301-620x.89b2.18576	197
96	Bozic KJ, Lau E, Kurtz S, Ong K, Rubash H, Vail TP, Berry DJ (2012) Patient-Related Risk Factors for Periprosthetic Joint Infection and Postoperative Mortality Following Total Hip Arthroplasty in Medicare Patients. Journal of Bone and Joint Surgery-American Volume 94A (9):794-800. doi:10.2106/jbjs. K.00072	194
97	Brodner W, Bitzan P, Meisinger V, Kaider A, Gottsauner-Wolf F, Kotz R (2003) Serum cobalt levels after metal-on-metal total hip arthroplasty. Journal of Bone and Joint Surgery-American Volume 85A (11):2168-2173. doi:10.2106/00004623-200311000-00017	194
98	Callaghan JJ, Albright JC, Goetz DD, Olejniczak JP, Johnston RC (2000) Charnley total hip arthroplasty with cement - Minimum twenty-five-year follow-up. Journal of Bone and Joint Surgery-American Volume 82A (4):487-497. doi:10.2106/00004623-200004000-00004	193
99	Siguier T, Siguier M, Brumpt B (2004) Mini-incision anterior approach does not increase dislocation rate - A study of 1037 total hip replacements. Clinical Orthopaedics and Related Research (426):164-173. doi:10.1097/01.blo.0000136651.21191.9f	191
100	Saleh K, Olson M, Resig S, Bershadsky B, Kuskowski M, Gioe T, Robinson H, Schmidt R, McElfresh E (2002) Predictors of wound infection in hip and knee joint replacement: results from a 20 year surveillance program. Journal of Orthopaedic Research 20 (3):506-515. doi:10.1016/s0736-0266(01)00153-x	191

**Fig. 4:**
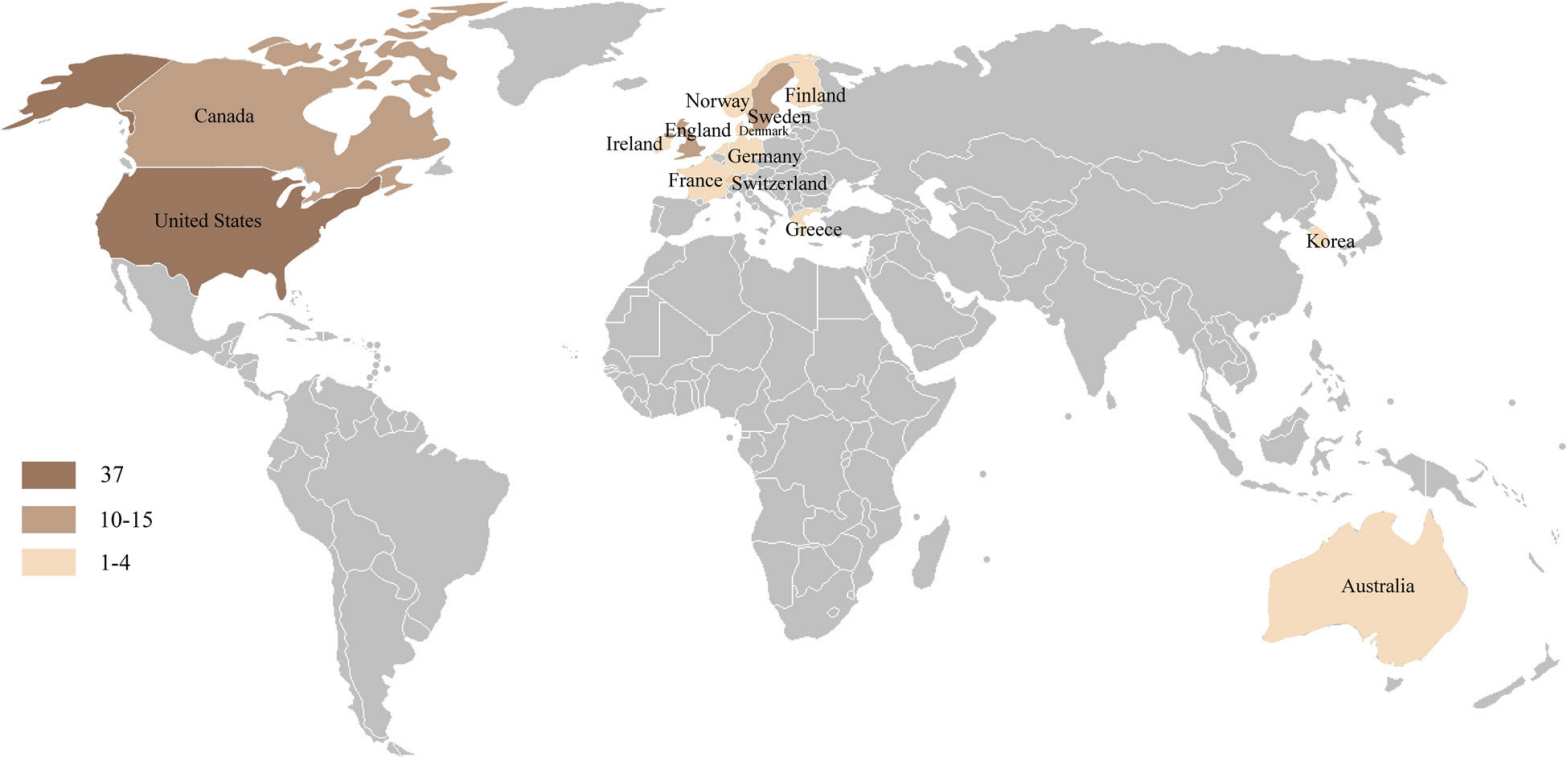
Geographical distribution of all articles. The map showed that almost all of the articles came from two regions: North America and Western Europe. Only 3 articles scattered in other areas: Australia and South Korea

These articles were distributed in 15 countries, led by the USA (*n* = 37) followed by Sweden (*n* = 15), England (*n* = 14), Canada (*n* = 11), and so on. The distribution is demonstrated on the world map (Fig. 4). Almost all of the articles came from two regions: North America and Western Europe. Only 3 articles scattered in other areas: Australia and South Korea. The articles from the USA were mainly published in The Journal of Bone and Joint Surgery-American Volume and articles from the UK were mainly published in The Journal of Bone and Joint Surgery-British Volume.

All of the articles were published in 21 journals. Thirty-six articles were published in The Journal of Bone and Joint Surgery-American Volume, followed by The Journal of Bone and Joint Surgery-British Volume (*n* = 15), Clinical Orthopaedics and Related Research (*n* = 9), Lancet (*n* = 5), New England Journal of Medicine (*n* = 5), and the rest of them as shown in (Table 2).

**Table 2:** Journal in which the top-cited 100 articles were published

Journal	No. of articles
Journal of Bone and Joint Surgery American volume	36
Journal of Bone and Joint Surgery British volume	15
Clinical Orthopaedics and Related Research	9
Lancet	6
New England Journal of Medicine	5
Journal of Orthopaedic Research	4
Acta orthopaedica Scandinavica	3
Archives of internal medicine	2
Jama	2
The Journal of arthroplasty	3
Thrombosis and Haemostasis	2
Journal of Rheumatology	2
Journal of Thrombosis and Haemostasis	2
Acta Orthopaedica	1
American Journal of Medicine	1
Arthritis and Rheumatism	1
Bmc Musculoskeletal Disorders	1
BMJ (Clinical research ed)	1
Circulation	1
Journal of Biomechanics	1
Journal of biomedical materials research	1
Journal of Clinical Pharmacology	1

The first author with the most articles and their basic information is listed in Table 3. Eriksson BI, from Sweden, had 7 fist authorships mainly in the field of thrombogenesis. The total citations of his selected articles were 3209.

**Table 3:** List of first authors with frequent articles within the top-cited list

Frequent first authors	No. of articles	Author’s Affiliation
Eriksson BI	7	Sahlgrenska Univ Hosp Ostra, Dept Orthopaed, SE-41685 Gothenburg, Sweden
Jones CA	2	Univ Alberta, Fac Pharm, Dept Publ Hlth Sci, Edmonton, AB T6G 2 N2, Canada
Mahomed NN	2	Univ British Columbia, Dept Orthopaed, Vancouver, BC 45Z 4E3, Canada
Martell JM	2	Department of Orthopaedic Surgery, Rush-Presbyterian-St. Luke's Medical Center, Chicago, Illinois 60,612, USA

**Fig. 5:**
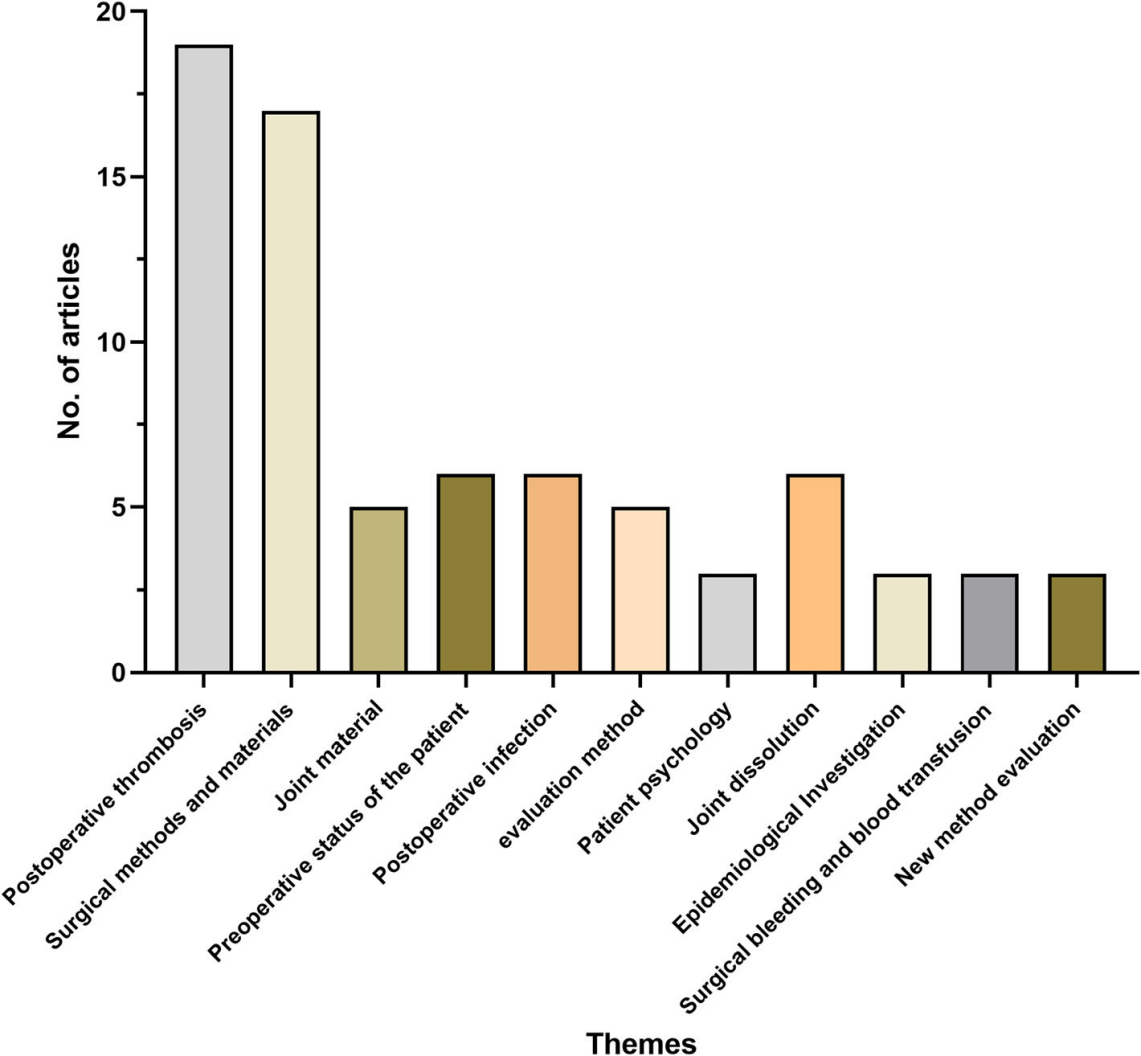
The themes distribution of all the articles. The most mentioned theme was postoperative thrombosis (*n* = 19), followed by surgical methods and materials (*n* = 17)

**Fig. 6:**
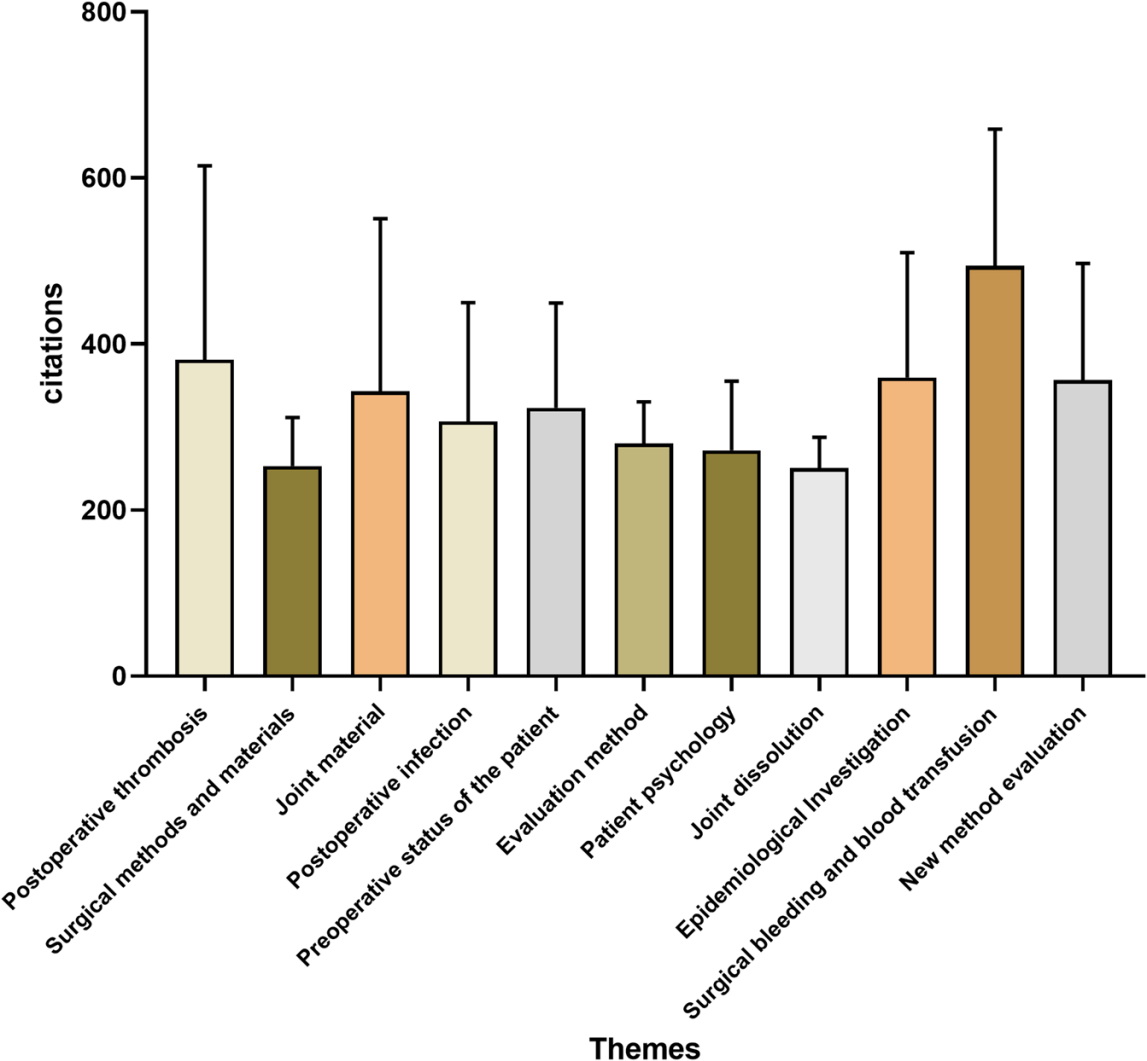
Mean citation per article based on theme. N. S not significant. One-way ANOVA revealed no significant difference in citations per article among the various themes (*P* = 0.296)

The top 100 most cited articles focused principally on the following themes: postoperative thrombosis (*n* = 19), surgical methods and materials (*n* = 17), joint materials (5), preoperative status of the patient (*n* = 6), postoperative infection (*n* = 6), joint dislocation (*n* = 6), evaluation method (*n* = 5), patient psychology (*n* = 3), epidemiological investigation (*n* = 3), surgical bleeding and blood transfusion (*n* = 3) and new technique evaluation (*n* = 3) (Fig. 5). The most mentioned theme was postoperative thrombosis (*n* = 19), followed by surgical methods and materials (*n* = 17). One-way ANOVA revealed no significant difference in citations per article among the various themes (*P* = 0.296) (Fig. 6).

**Fig. 7:**
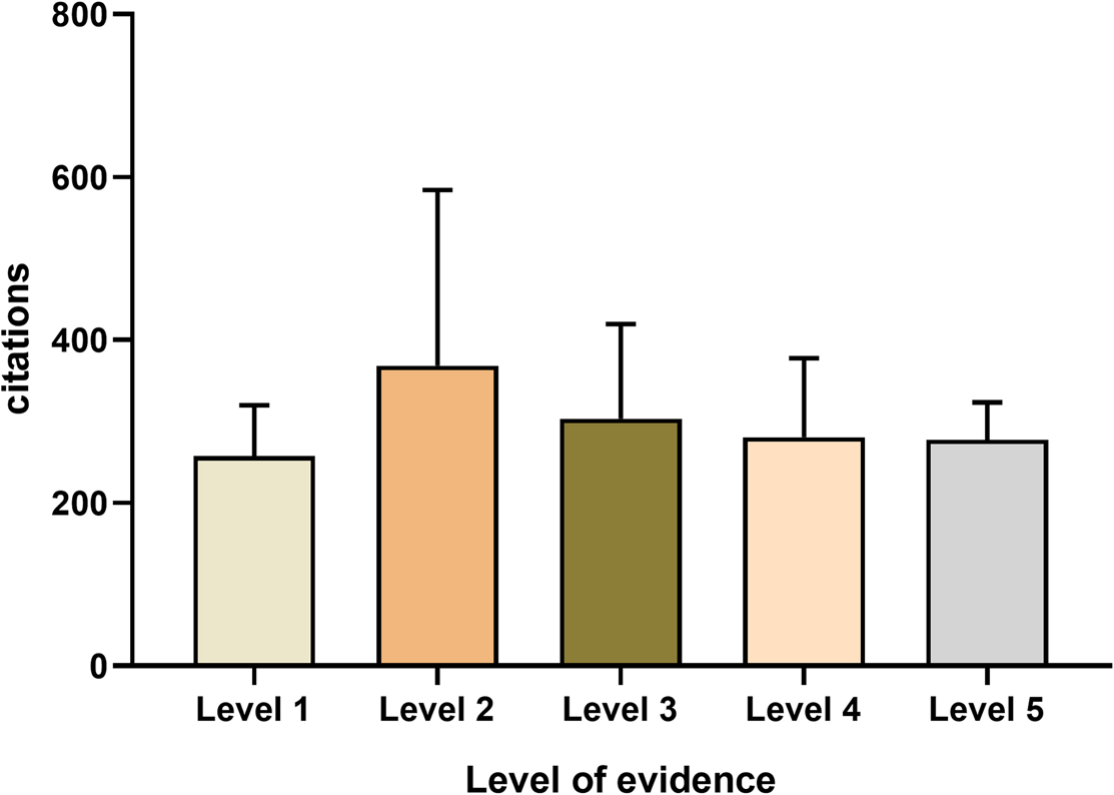
Mean citation per article based on level of evidence. N. S not significant. No significant difference in citations per article among the various levels of evidence has been found by using the one-way ANOVA

The largest number of articles were level III evidence (*n* = 35), with a mean number of 304 ± 116 citations per article, followed by the number of articles which represented level II (*n* = 25) and level IV (*n* = 25), with a mean number of 269 ± 216 and 281 ± 97 respectively. No significant difference in citations per article among the various levels of evidence has been found by using the one-way ANOVA (Fig. 7).

**Fig. 8:**
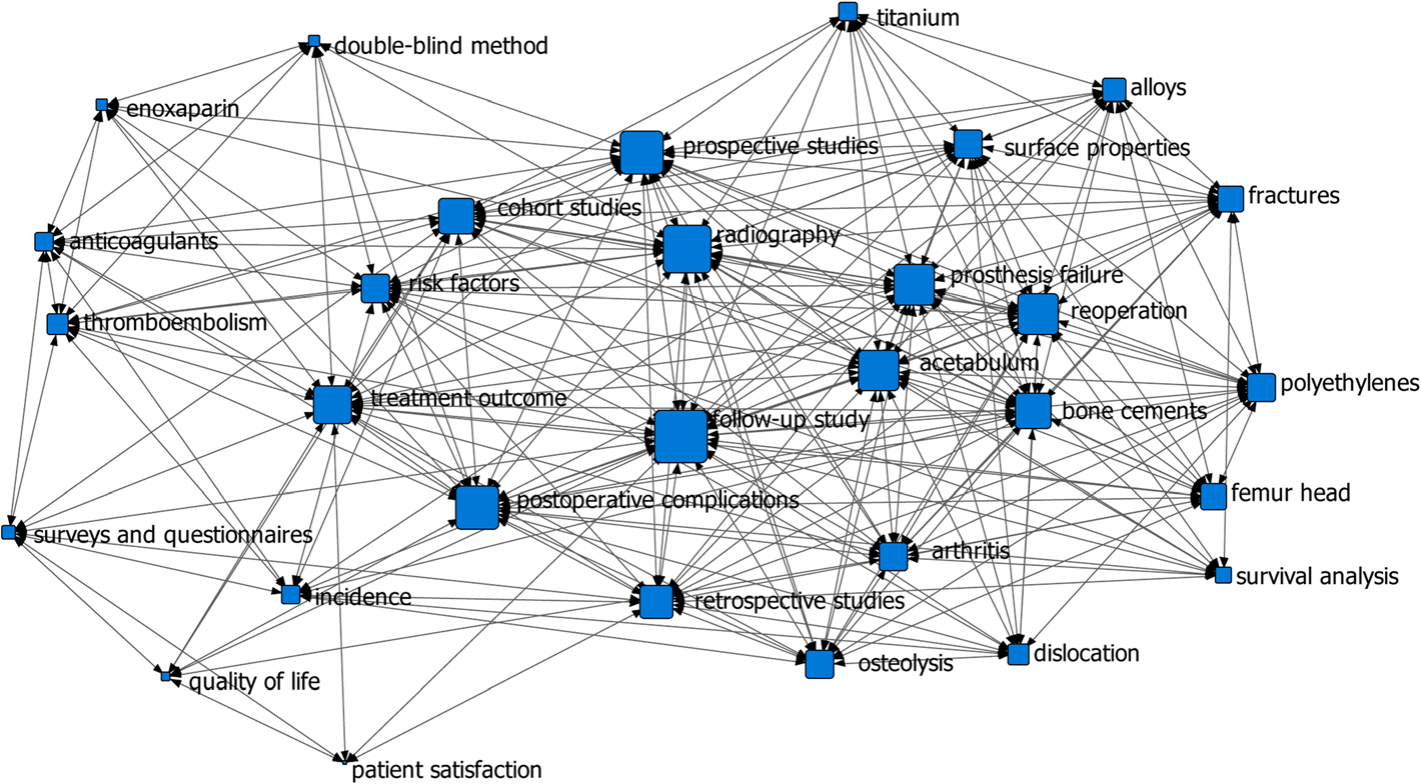
Degree centrality analysis in the 1990s (46 articles). It showed that “follow-up study, radiography, acetabulum, reoperation, and bone cements” had a high degree of centrality in the 1990s

**Fig. 9:**
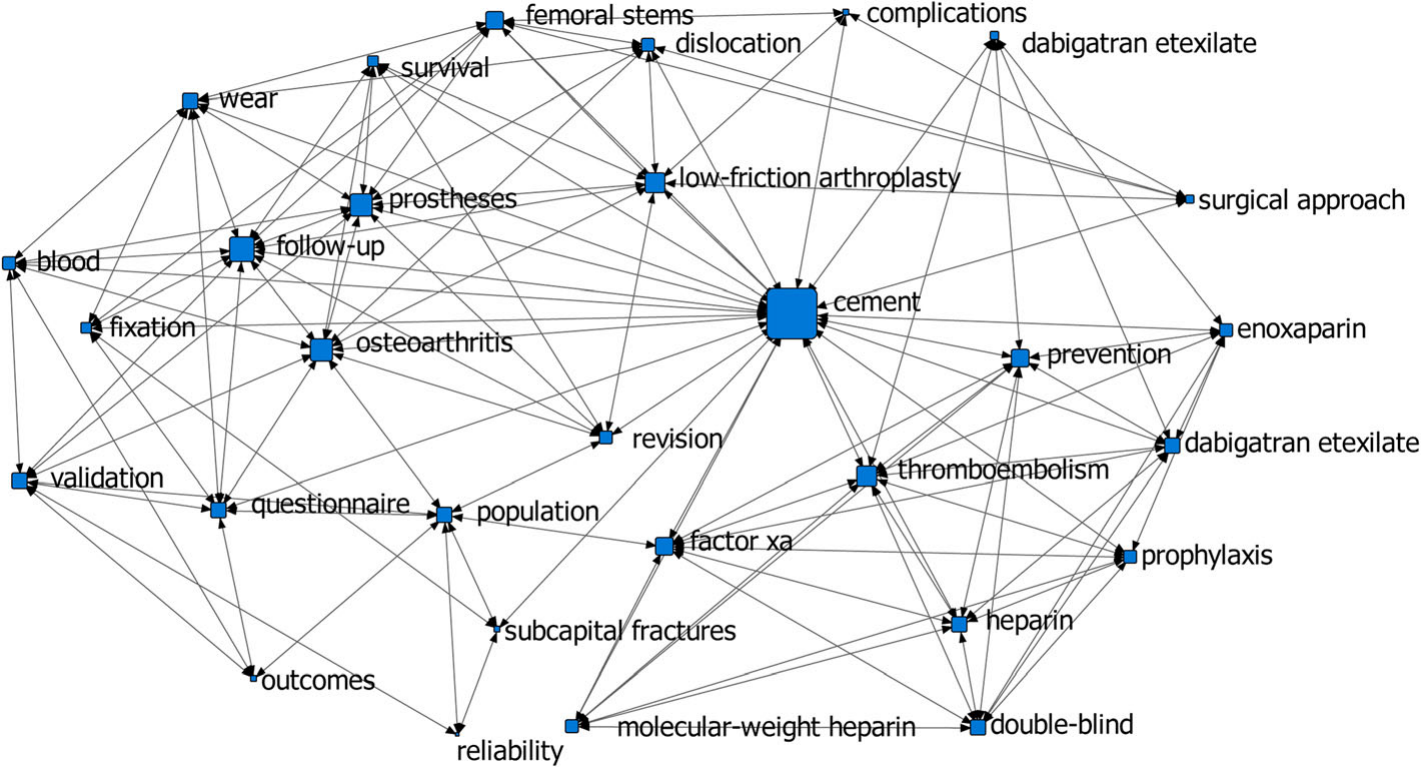
Degree centrality analysis in the 2000s and 2010s (54 articles). It showed that “cement” had a high degree of centrality in the 2000s and 2010s

Network analysis of the author’s key words or subject terms has been done in two periods of article published time: in the 1990s (46 articles) and in the 2000s and 2010s (54 articles). The result indicated that “follow-up study, radiography, acetabulum, reoperation, and bone cements” had a high degree of centrality in the 1990s (Fig. 8), while “cement” had a high degree of centrality in the 2000s and 2010s (Fig. 9).

## Discussion

This is the first bibliometric analysis of total hip arthroplasty papers. Several significant findings can be drawn from this analysis of the top 100 most cited papers published on total hip arthroplasty. The maximum number of citations only reached 994, and this was a paper on anticoagulation. One explanation is that the current analysis only focuses on articles published from 1990 to 2019. Therefore, there may not be enough time for the articles to be thoroughly cited. Majority of the top-cited articles were published in the 1990s with a similar number in the 2000s and only 5 articles published since 2010 made the top 100 most cited. A phenomenon known as “obliteration by incorporation” [15] was not demonstrated in our analysis. The regional distribution of articles has also been demonstrated. Majority of the articles originated from two major regions: North America and Western Europe. There are several reasons for this phenomenon: (1) total hip arthroplasty surgery originated in the USA in the 1960s; hence, they started to study it earlier and in greater detail; (2) Sweden was the first country to establish an artificial joint registry and they continue to analyze the data annually, Finland, Norway, and Denmark also established a joint registry system increasing their publications; (3) countries such as the USA, England, and Sweden have a fairly developed economy to support medical research; it has previously been demonstrated that there is a weak correlation between the gross domestic product (GDP) per capita of a country and their research achievements [16]. It is of great importance that the achievements in these countries are shared worldwide so that everyone can benefit from their research advances.

This article also examined the theme distribution of the most cited articles. Surgical methods and materials, and postoperative thrombosis were the top two. The former has always been a research hotspot since the artificial joint replacement was invented [17–19]. Researchers share advances in how they improve joint stability and surgical prognosis through modifying the artificial joint or changing the materials. The degree of centrality analysis of the author’s key words also indicated that “cement” has a high degree of centrality both in the 1990s and again in the 2000s. Using or not using cement during operation is still a controversial issue [20–22], which needs to be further explored in the future. Postoperative thrombosis has also been at the center of research interest [23]. Postoperative thrombosis is one of the important complications following total hip arthroplasty, which has a great impact on the prognosis of patients. In fact, blood clots that form after surgery have been of interest to researchers since very early years [24–27]. Up to now, thrombus formation after surgery is still widely investigated in different research fields including hip arthroplasty [28–30].

The level of evidence analysis demonstrated that the level of evidence ranged between I and IV, mainly level II, III, and IV. These are relatively high and evenly distributed, which is different from other bibliometric studies [31, 32]. This may be because researchers have invested more in total hip arthroplasty study in the past 30 years because it is a significant advancement in the treatment of pain and morbidity and has become the focus of people’s attention. In the future, with the increasing demand for quality of life and the increasing problem of an aging population in some countries, especially in China, it is expected that total hip arthroplasty surgery will increase more rapidly, as will the related research articles. Older people are more likely to have hip problems; therefore, the aging population issue will promote the development of total hip replacement in these countries.

Of all the articles selected, the top three cited were all about post-operative thrombosis prevention. In first place is the research of Eriksson, BI. et al., published in the New England Journal of Medicine, which compared the effect of rivaroxaban and enoxaparin on preventing postoperative thrombosis. The results showed that in total hip replacement patients, once-daily oral administration of 10 mg rivaroxaban was more effective in preventing thrombosis than once-daily subcutaneous injection of 40 mg enoxaparin [33]. The article with the second most citations is another research article by Eriksson, BI. et al., published in the Lancet. They compared the efficacy of dabigatran etexilate and enoxaparin in preventing thrombosis after total hip surgery. It has been proved that oral dabigatran etexilate is as effective and safe as enoxaparin in reducing the risk of venous thromboembolism after total hip replacement [34].

Actually, in the preliminary analysis, articles published on total hip arthroplasty were included from 1950 to 2019. Not surprisingly, the result showed that most of the top-cited articles were about surgical materials and prognosis. The most cited article was a study by DeLee, J G et al. in 1976, which reached 2289 citations. They explored the radiological separation of acetabular cement junctions after total hip arthroplasty and the result showed that the application of cement was not the explanation for the demarcation of the cement-bone junction; therefore, they concluded that there is no fundamental defect in the principle of using cement for acetabular components [35]. This research was of great importance in promoting the use of cement in total hip arthroplasty. Although these articles are of great significance, the current bibliographic analysis aimed to focus on the most important advances in total hip arthroplasty over the last 30 years. From the 1990s onwards, there were more and more articles published on postoperative thromboembolism prevention, and they were becoming more topical and influential.

This analysis yielded some valuable information, but there were limitations. While articles were filtered by how much they were cited, which overlooks the newly published articles that are significant in this field but have yet to reach high citation levels. Hence, it is biased towards historical articles. At the same time, only published articles were included while meeting records, textbooks, meta-analysis and reviews were excluded, which may result in omission bias.

## Conclusion

This article highlights the top 100 most cited articles in the total hip arthroplasty over the last 30 years, including their time and area of distribution, their research topic, their authorship as well as their level of evidence. This analysis sheds light on the spotlights and characteristics of total hip arthroplasty research since 1990 to 2019. Furthermore, we have noticed that, currently, postoperative thromboembolism plays a major role in the field of total hip arthroplasty. However, no one has really solved this tough problem. Meanwhile, most of them were focus on the clinical trial while the basic laboratory research was insufficient, which calls for more researches based on this topic. Thus, in the future, the prevention and management of thrombosis after total hip arthroplasty should be further investigated, especially the basic mechanisms.

## Data Availability

All data generated or analyzed during this study are included in this published article.

## Abbreviations

ANOVA: Analysis of variance
GDP: Gross domestic product

## References

[1] Cabanela ME (1990) Bipolar versus total hip arthroplasty for avascular necrosis of the femoral head. A comparison. Clin Orthopaedics Related Res (261):59-62. 2245563

[2] Pang Y, Zheng X, Pei F, Chen Y, Guo K, Zhao F (2019). A retrospective study to compare the efficacy and postoperative outcome of total hip arthroplasty with internal screw fixation in patients with avascular necrosis of the femoral head. Med Sci Monitor.

[3] Fehrle MJ, Callaghan JJ, Clark CR, Peterson KK (1993). Uncemented total hip arthroplasty in patients with aseptic necrosis of the femoral head and previous bone grafting. J Arthroplast.

[4] Ritter MA, Carr K, Herbst SA, Eizember LE, Keating EM, Faris PM, Meding JB (1996). Outcome of the contralateral hip following total hip arthroplasty for osteoarthritis. J Arthroplast.

[5] Aminian K, Trevisan C, Najafi B, Dejnabadi H, Frigo C, Pavan E, Telonio A, Cerati F, Marinoni EC, Robert P, Leyvraz PF (2004). Evaluation of an ambulatory system for gait analysis in hip osteoarthritis and after total hip replacement. Gait Posture.

[6] Dougados M (2002). Requirement for total hip arthroplasty: an outcome measure of hip osteoarthritis. Presse Med.

[7] Johansson T, Jacobsson SA, Ivarsson I, Knutsson A, Wahlstrom O (2000). Internal fixation versus total hip arthroplasty in the treatment of displaced femoral neck fractures - a prospective randomized study of 100 hips. Acta Orthop Scand.

[8] Watson D, Bostrom M, Salvati E, Walcott-Sapp S, Westrich G. Primary total hip arthroplasty for displaced femoral neck fracture. Orthopedics. 2008;31(10):990. 19226015

[9] She GR, Chen JY, Zhou ZQ, Zha ZG, Liu N (2017). Total hip arthroplasty for femoral neck fracture with pyoderma gangrenosum patient: a case report. Int J Surg Case Rep.

[10] Chiu W-T, Ho Y-S (2007). Bibliometric analysis of tsunami research. Scientometrics.

[11] Pena-Ibanez F, Ruiz-Iniguez R (2019). The most cited articles in Spanish nursing (1997-2016): a bibliometric analysis. Rqr Enfermeria Comunitaria.

[12] Ponce Suarez VN, Perez Sousa H, Sanchez del Collado A (2019). Publication trends of the journal Cuba (1962-1969): a bibliometric analysis. Bibliotecas-Anales De Investigacion.

[13] Bhandari M, Swiontkowski MF, Einhorn TA, Tornetta P, Schemitsch EH, Leece P, Sprague S, Wright JG (2004). Interobserver agreement in the application of levels of evidence to scientific papers in The American Volume of The Journal of Bone and Joint Surgery. J Bone Joint Surgery-American Volume.

[14] Borgatti SP, Everett MG, Freeman LC (2002). Ucinet for windows: software for social network analysis.

[15] Ahmad SS, Ahmad SS, Kohl S, Ahmad S, Ahmed AR (2015). The hundred most cited articles in bariatric surgery. Obes Surg.

[16] Tao Tianzhu, Zhao Xiaohong, Lou Jingsheng, Bo Lulong, Wang Fei, Li Jinbao, Deng Xiaoming (2012). The top cited clinical research articles on sepsis: a bibliometric analysis. Critical Care.

[17] Iyer LS, Jayasekaran T, Blunck CF, Selvam RP (1984). Development of optimized epoxy graphite implant for the total hip joint. ISA Trans.

[18] Breck LW, Peltier LF (2005). The classic: metal to metal total hip joint replacement using the Urist socket: an end result study. 1973. Clin Orthop Relat Res.

[19] Breck LW. Metal to metal total hip joint replacement using the Urist socket. An end result study. 1973. Clin Orthopaedics Related Res. 1992;(285):4–6. 1446452

[20] Wroblewski BM (1993). Cementless versus cemented total hip arthroplasty. A scientific controversy?. Orthop Clin North Am.

[21] Tanzer M, Graves SE, Peng A, Shimmin AJ (2018). Is cemented or cementless femoral stem fixation more durable in patients older than 75 years of age? A comparison of the best-performing stems. Clin Orthop Relat Res.

[22] Assaf A, Manara JR, Teoh KH, Evans AR (2019). Mid-term clinical results of the cementless R3 cup and Polarstem total hip arthroplasty. Eur J Orthopaedic Surg Traumatol.

[23] Swayze OS, Nasser S, Roberson JR (1992). Deep venous thrombosis in total hip arthroplasty. Orthopedic Clin North Am.

[24] Homans J (1945). The surgery of the veins of the legs; varicosity and some problems in thrombosis. Rhode Island Med J.

[25] Smith GVS, Mulligan WJ (1948). Dicumarol prophylaxis against venous thrombosis in women undergoing surgery. Surg Gynecol Obstet.

[26] Pratt JP, Hodgkinson CP, McConnell FG (1951). Prevention of thrombosis and embolism in gynecological surgery. Am J Obstet Gynecol.

[27] Merei L (1951). Surgery in 3 cases of jugular thrombosis extending to the subclavian vein (Adatok a clavicula ala (vena anonymaig) terjedo jugularisthrombosis kezelesehez.). Orv Hetil.

[28] Huang J (2019). Infection and thrombosis in cardiac surgery: is there a common ground?. J Cardiothorac Vasc Anesth.

[29] Suh J, Braseth A, Challa A, Enke T, Manatsathit W (2019). Outcomes of abdominal surgery in patients with portal vein thrombosis. Gastroenterology.

[30] Diaz Quintero LA, Fuentes HE, Tafur AJ, Majmudar K, Salazar Adum JP, Golemi I, Paz LH, Stocker S, Talamonti M (2019). Pancreatic cancer thromboembolic outcomes: rate of thrombosis after adenocarcinoma and non-adenocarcinoma pancreatic cancer surgery. Int Angiol.

[31] Namdari S, Baldwin K, Kovatch K, Huffman GR, Glaser D (2012). Fifty most cited articles in orthopedic shoulder surgery. J Shoulder Elb Surg.

[32] Ahmad Sufian S, Evangelopoulos Dimitrios S, Abbasian M, Röder Christoph, Kohl Sandro (2014). The Hundred Most-Cited Publications in Orthopaedic Knee Research. The Journal of Bone and Joint Surgery-American Volume.

[33] Eriksson BI, Borris LC, Friedman RJ, Haas S, Huisman MV, Kakkar AK, Bandel TJ, Beckmann H, Muehlhofer E, Misselwitz F, Geerts W, Grp RS (2008). Rivaroxaban versus enoxaparin for thromboprophylaxis after hip arthroplasty. N Engl J Med.

[34] Eriksson BI, Dahl OE, Rosencher N, Kurth AA, van Dijk CN, Frostick SP, Prins MH, Hettiarachchi R, Hantel S, Schnee J, Buller HR, Grp R-NS (2007). Dabigatran etexilate versus enoxaparin for prevention of venous thromboembolism after total hip replacement: a randomised, double-blind, non-inferiority trial. Lancet.

[35] DeLee JG, Charnley J (1976). Radiological demarcation of cemented sockets in total hip replacement. Clin Orthop Relat Res.

